# Supplementary material for: Pan-cancer classification of single cells in the tumour microenvironment
Source: Nat Commun. 2023 Mar 23;14:1615. doi: 10.1038/s41467-023-37353-8 (PMC10036554; doi:10.1038/s41467-023-37353-8)
Supplement: Supplementary file 1 — Supplementary Information [file 41467_2023_37353_MOESM1_ESM.docx]

**Supplementary Information for**

**Pan-cancer classification of single cells in the tumour microenvironment**

Ido Nofech-Mozes^1,2^, David Soave^1,3^, Philip Awadalla^1,2,4,^*, Sagi Abelson^1,2,^*

^1^ Ontario Institute for Cancer Research, Toronto, ON, Canada.

^2^ Department of Molecular Genetics, University of Toronto, Toronto, ON, Canada.

^3^ Department of Mathematics, Wilfrid Laurier University, Waterloo, ON, Canada.

^4^ Dalla Lana School of Public Health, University of Toronto, Toronto, ON, Canada.

*Correspondence to: [sagi.abelson@oicr.on.ca](mailto:sagi.abelson@oicr.on.ca) & [philip.awadalla@oicr.on.ca](mailto:philip.awadalla@oicr.on.ca)

These authors jointly supervised this work: Philip Awadalla, Sagi Abelson.

**Content:**

- **Supplementary Figures 1-12**
- **Supplementary Notes 1-2**
- **References**


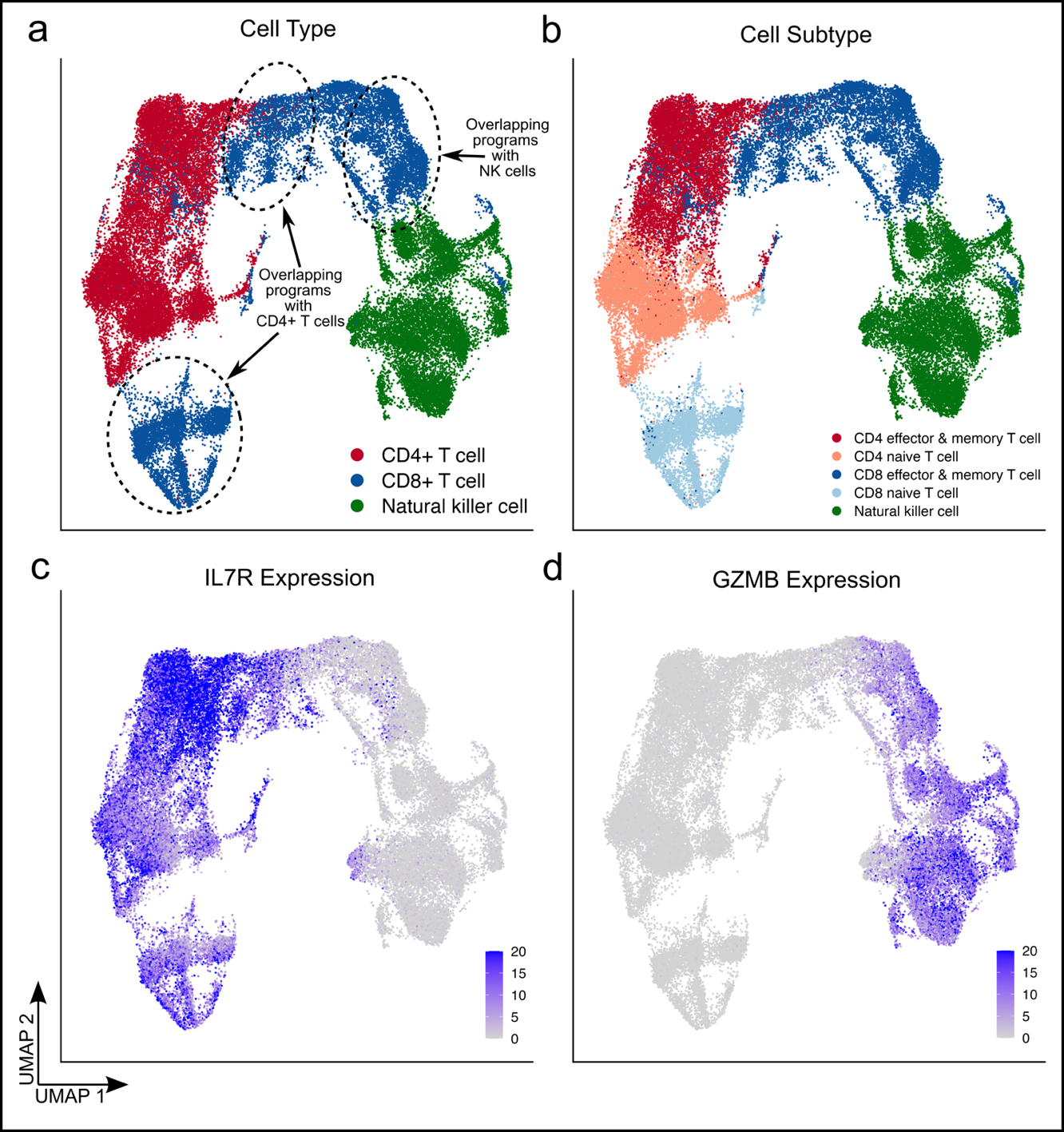


**Supplementary Fig. 1.** **Shared transcriptional programs among related non-malignant cell types.** Unsupervised clustering of CD8+ T cells, CD4+ T cells, and natural killer cells visualized on UMAP. Dots representing single cells coloured by **a**, cell type, **b**, cell subtype, **c**, IL7R expression, and **d**, GZMB expression. IL7R expression levels differentiate CD4+ T cells from NK cells, yet not from all CD8+ cells. High IL7R expression is observed in both CD4+ and CD8+ naïve cells. Similarly, GZMB expression levels differentiate NK cells from CD4+ T cells yet not from all CD8 cells. High GZMB expression is observed in both NK and CD8 cells with cytotoxic functions.

**
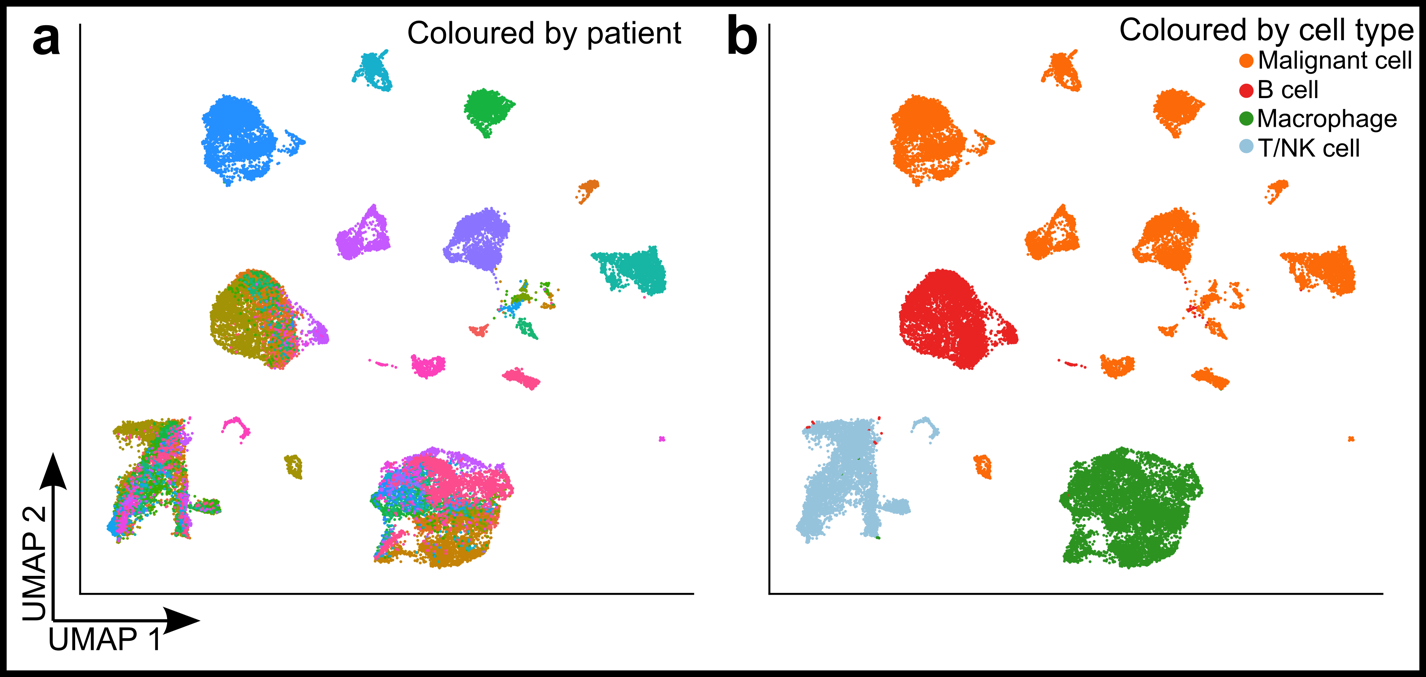
Supplementary Fig. 2. Complexity associated with interpatient tumour cell heterogeneity.**

Unsupervised clustering of 17 lung cancer biopsies^1^ reveals high heterogeneity in patient-specific malignant cells. In comparison, non-malignant cells show less extensive transcriptomic heterogeneity. Dots represent single cells coloured by **a,** patient ID, and **b,** cell type.


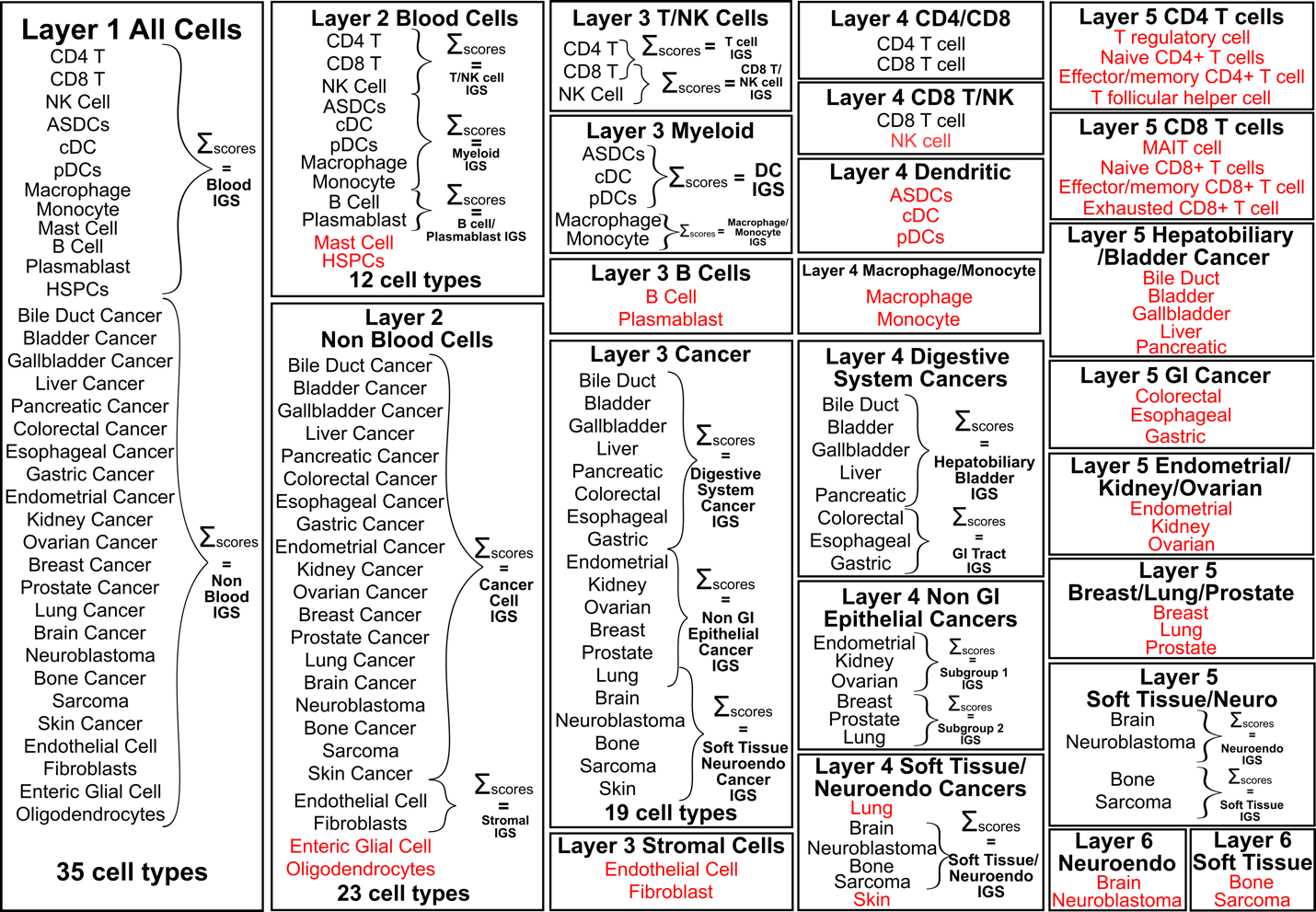


**Supplementary Fig. 3. Detailed overview of classification branches and intermediate-group scores** **within the core scATOMIC algorithm.** Random forests classification models representing different parental nodes (n=24) are used to derive scores for terminal cell types. Broad cell type intermediate-group scores are calculated by taking the sum of random forest scores for each terminal cell type indicated. Red text represents cell classes that can reach terminal classification in each model.


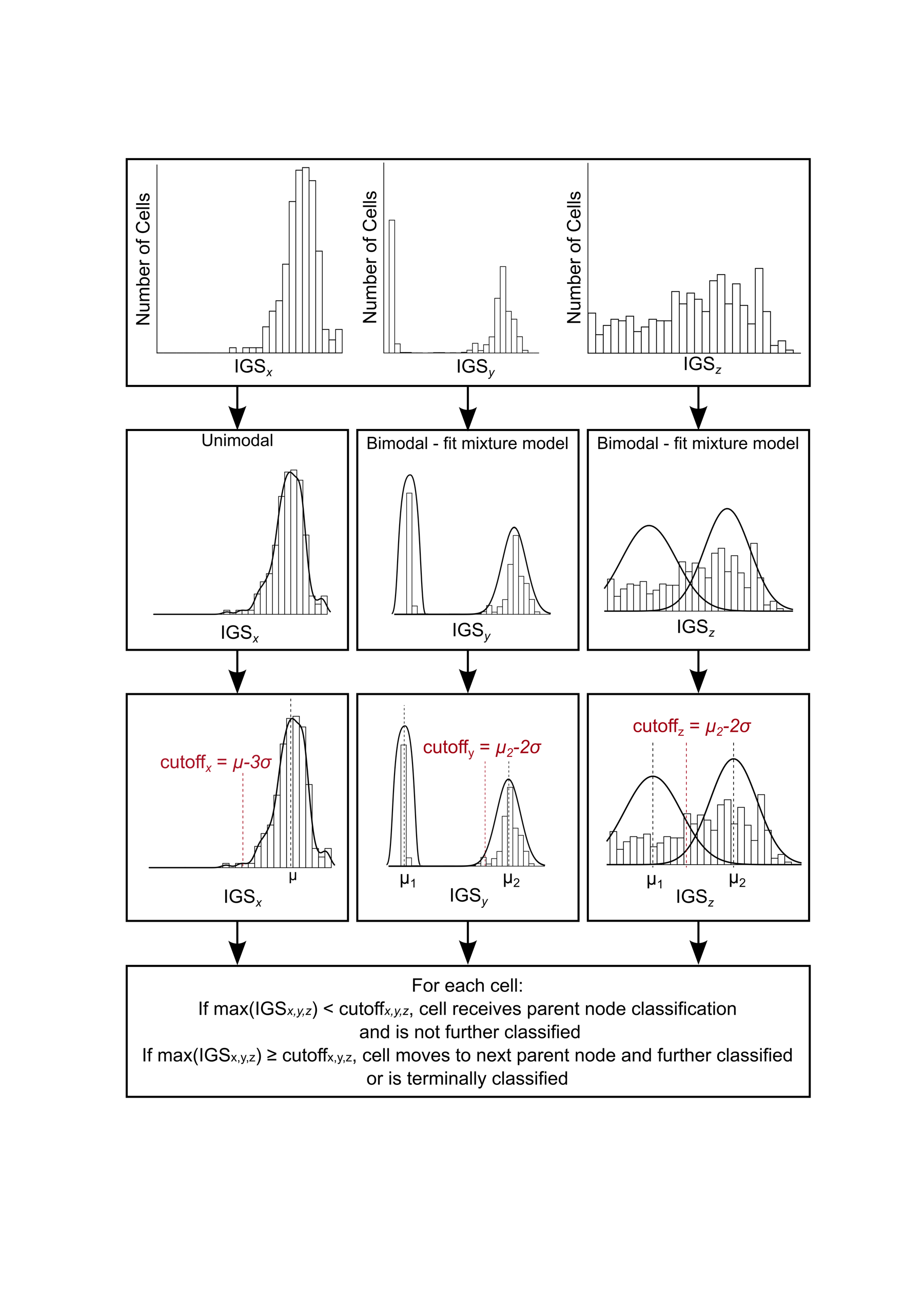


**Supplementary Fig. 4. Setting automatic IGS cut-offs at each classification branch.** Intermediate group score (IGS) cut-offs are determined based on the distribution of scores derive from all the cells being queried in each particular classification task. The modality of IGS distributions is classified as either unimodal or bimodal. For unimodal distributions the cut-off to associate one cell with a cell class is set to be three standard deviations (*σ*) from the mean (*µ*). For bimodal distributions scATOMIC fits a mixture model and estimates parameters using the expectation maximization algorithm. The cut-off is set to 2 standard deviations from the highest estimated mean (*µ*_2_). IGSx, IGSy, IGSz represent possible distributions of scores obtained from different models. For example, unimodal distribution can be seen when highly purified cell population is being interrogated by a particular model. 2-mode bimodal distribution is typical for the first classification task (i.e., Blood or Non-blood) of cancer TMEs.


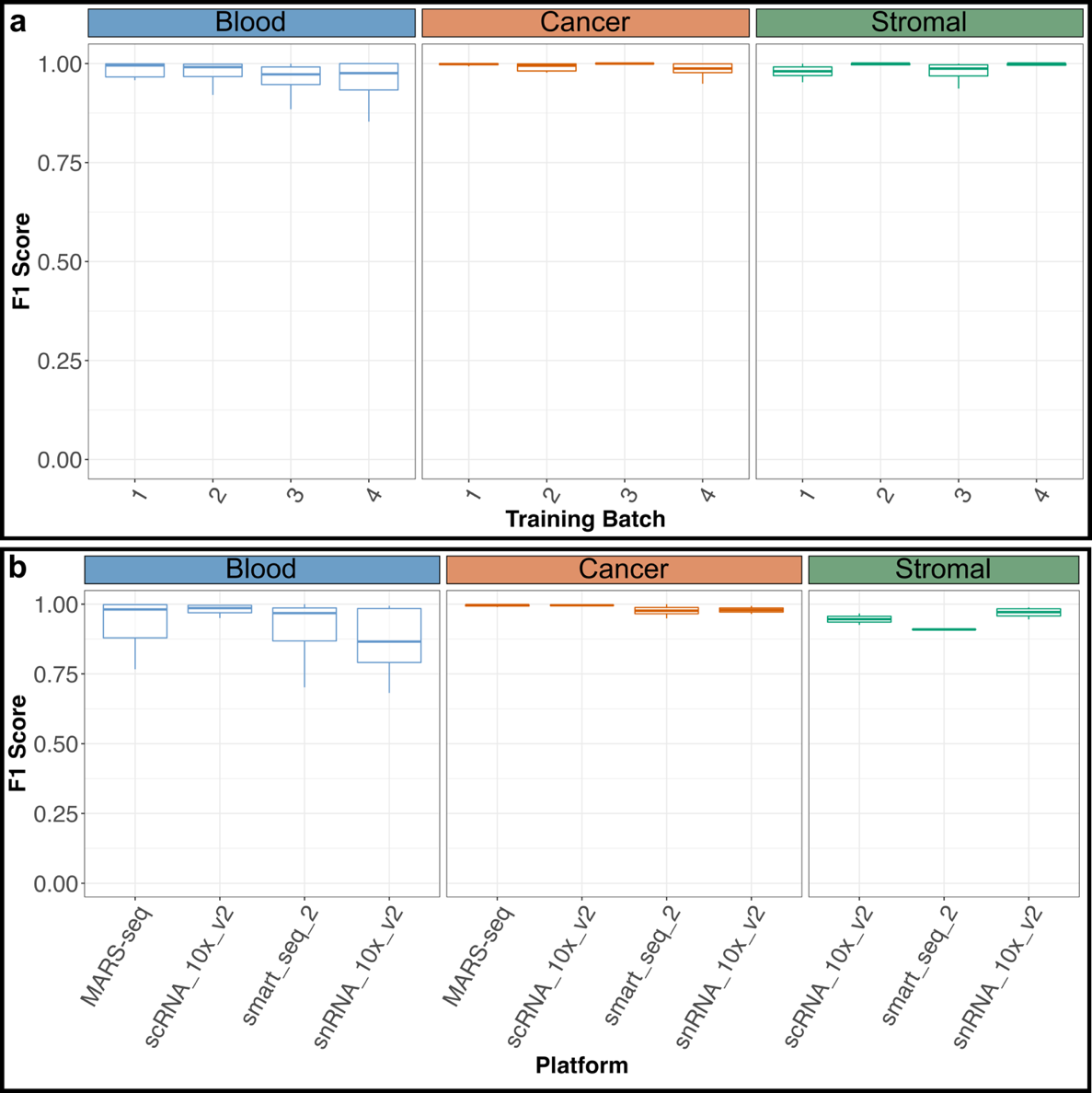


**Supplementary Fig. 5. scATOMIC performance evaluation with respect to technical effects.**

F1 scores are plotted across iterations for cells grouped under their corresponding category. **a,** Four iterations of scATOMIC were trained with sized matched technical batches and tested on cells from batches held out from training (training batch 1: n=18,219 cells held out for testing from 13 individuals, training batch 2: n=65,128 cells held out for testing from 32 individuals, training batch 3: n=38,311 cells held out for testing from 23 individuals, training batch 4: n=65,783 cells held out for testing from 24 individuals) . Insignificant differences in performance across batches are observed (Kruskal-Wallis test: P-value = 0.106, maximum difference in medians among blood, cancer, stromal = 0.023, 0.012, 0.019, respectively). **b,** scATOMIC performance was evaluated in training-independent melanoma samples profiled with different scRNA-seq platforms (MARS-seq: n=28,165 cells, scRNA-seq 10X V2: n=19,661 cells, Smart-seq2: n=4,610 cells, snRNA-seq: n=7,958 cells). F1 scores were not significantly different across platforms (Kruskal-Wallis test: P-value = 0.537, maximum difference in medians among blood, cancer, stromal = 0.120, 0.020, 0.062, respectively). For all plots, boxes and whiskers represent the lower fence, ﬁrst quartile (Q1), median (Q2), third quartile (Q3), and upper fence. Source data are provided as a Source Data file.


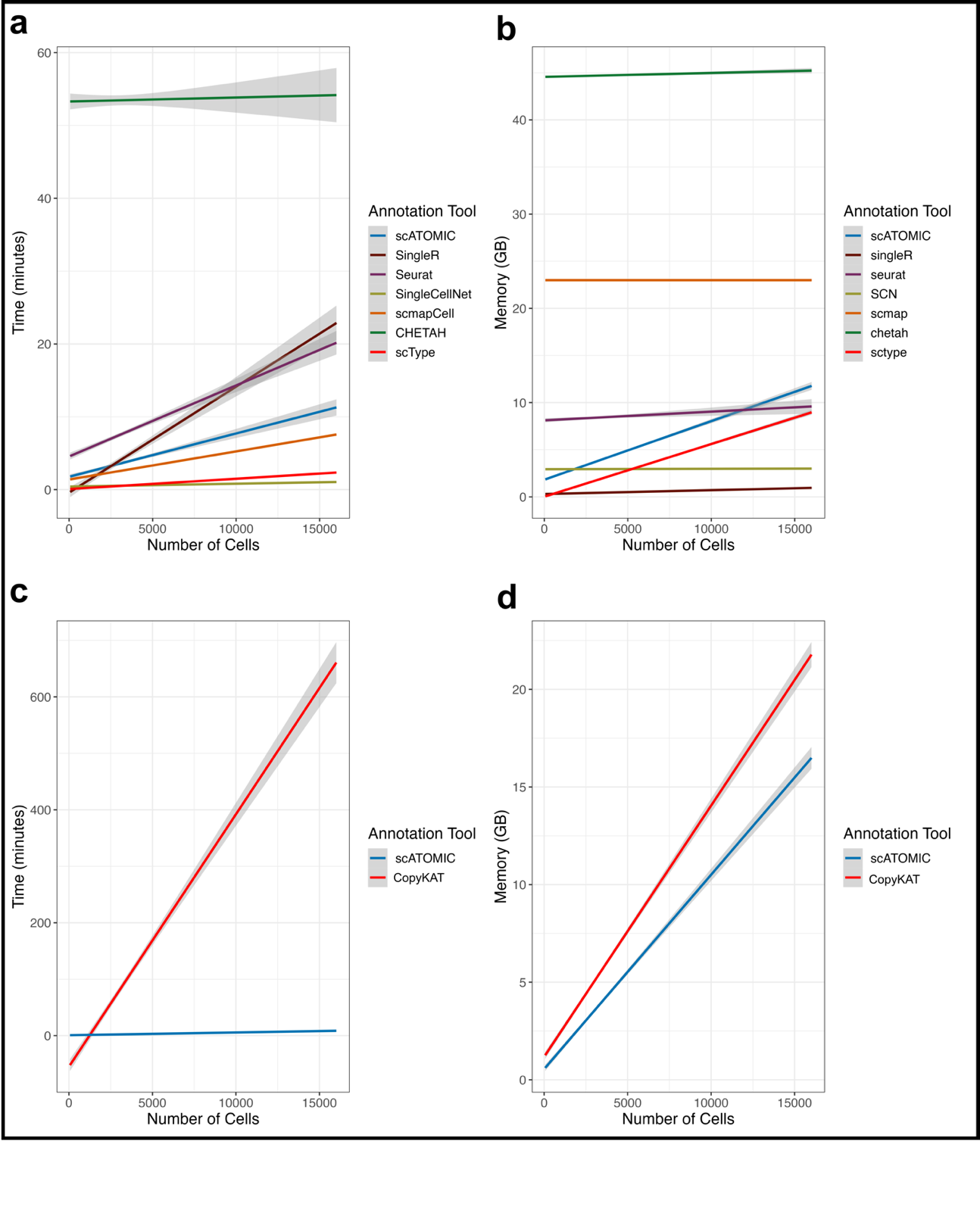


**Supplementary Fig. 6. Time and memory usage compared across classification methods.**

The time (**a**) and memory usage (**b**) to run scATOMIC cell type classification is compared to other cell type classifiers across the external validation datasets. The time (**c**) and memory usage (**d**) to run scATOMIC cancer signature scoring is compared to copy number variation (CNV) inference using CopyKAT across the external validation datasets. Colours represent the different tested annotation methods. For all methods, time to generate references or pretrained classifiers was not considered. All error bands (shaded areas) represent 95% confidence intervals. Source data are provided as a Source Data file.

**
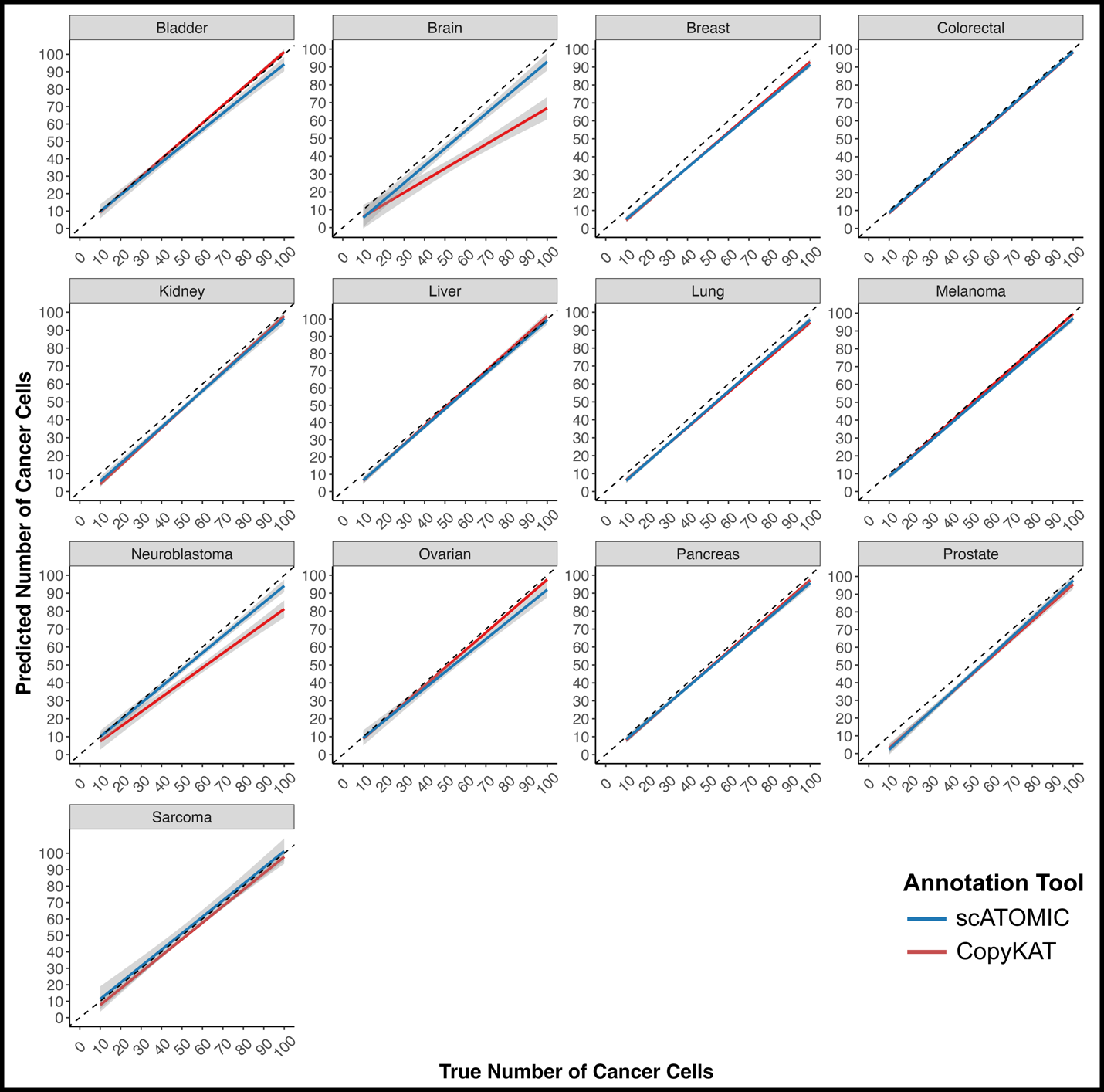
**

**Supplementary Fig. 7. In silico serial dilution of cancer cells.** Cancer cells were computationally diluted from 100 to 10 cancer cells per sample. The predicted number of cancer cells by scATOMIC (blue) and CopyKAT (red) versus the true number of cancer cells across all samples in the external validation is shown for each cancer type. Dashed lines represent a perfect 1:1 relationship. All error bands (shaded areas) represent 95% confidence intervals. Source data are provided as a Source Data file.


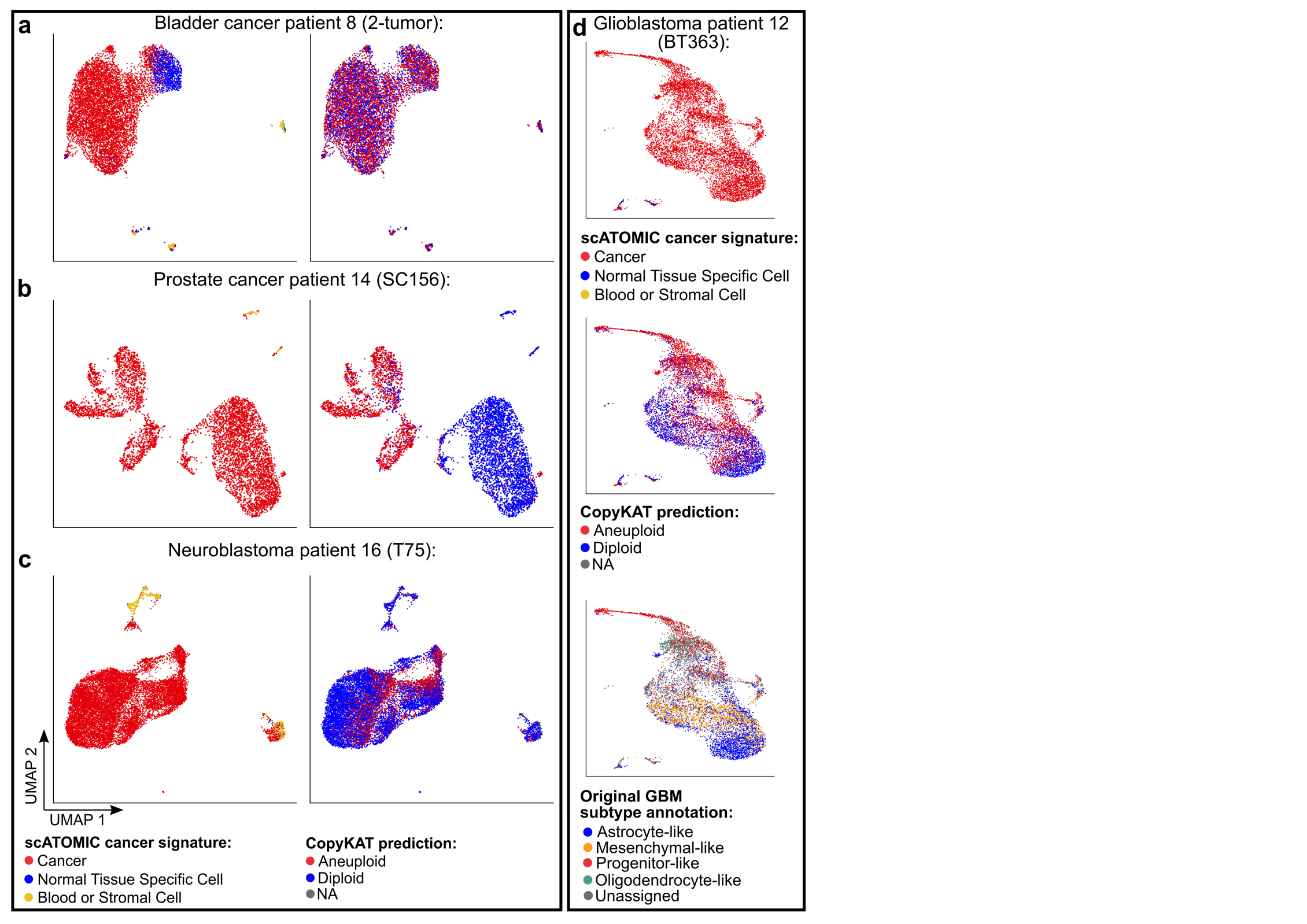


**Supplementary Fig. 8. Examples of discordant scATOMIC malignant status and CNV-based inferred ploidy.**  Cells are coloured according to scATOMIC malignant annotation and CopyKAT inferred copy number variation (CNV) status. **a,** A bladder tumour reported to have low CNV burden^2^. **b,** A prostate tumour specimen with few reference cells. The specimen reported to have 80% cancer cellularity^3^. **c,** A neuroblastoma tumour with an un-patterned prediction of aneuploid cells suggesting weak CNV profile intensities above the reference^4^. **d,** A glioblastoma tumour with multiple tumour cell populations^5^.

**
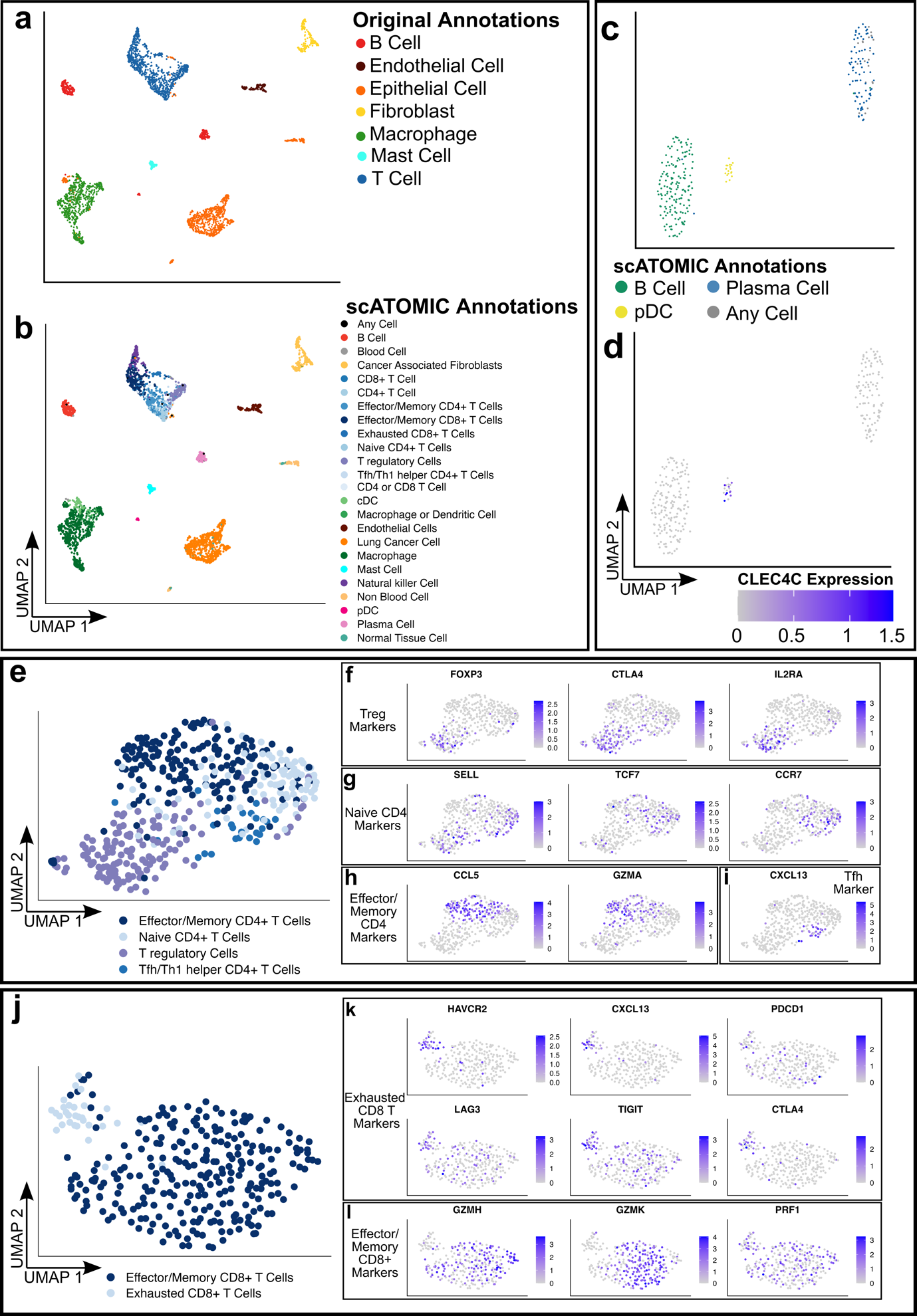
**

**Supplementary Fig. 9. Increasing the cellular resolution with scATOMIC’s core predictions.** scATOMIC was applied to a published lung cancer biopsy dataset^6^. Cells were clustered and visualized using the standard Seurat workflow and colored by **a**, the original published annotations^6^ and **b**, scATOMIC predictions. **c,** Cells that were originally annotated as B cells are illustrated on two UMAP dimensions. Single cells are labelled by scATOMIC annotations. scATOMIC separated B cells into B cells, Plasma Cells, and pDCs. **d,** Single cells are labelled by the expression level of the pDCs marker *CLEC4C*. CD4+ T cells were re-clustered and illustrated on two UMAP dimensions. Single cells are labelled by **e,** their scATOMIC-predicted CD4+ T state, **f,** T regulatory cell markers, **g,** naive CD4+ T cell markers, **h,** effector/memory CD4+ T cell markers, **i,** T follicular helper markers. CD8+ T cells were re-clustered and illustrated on two UMAP dimensions. Single cells are labelled by **j,** their scATOMIC-predicted CD8+ T state, **k,** exhausted CD8+ T cell markers, **l,** effector/memory CD8+ T cell markers. Expression values represent normalized read counts.


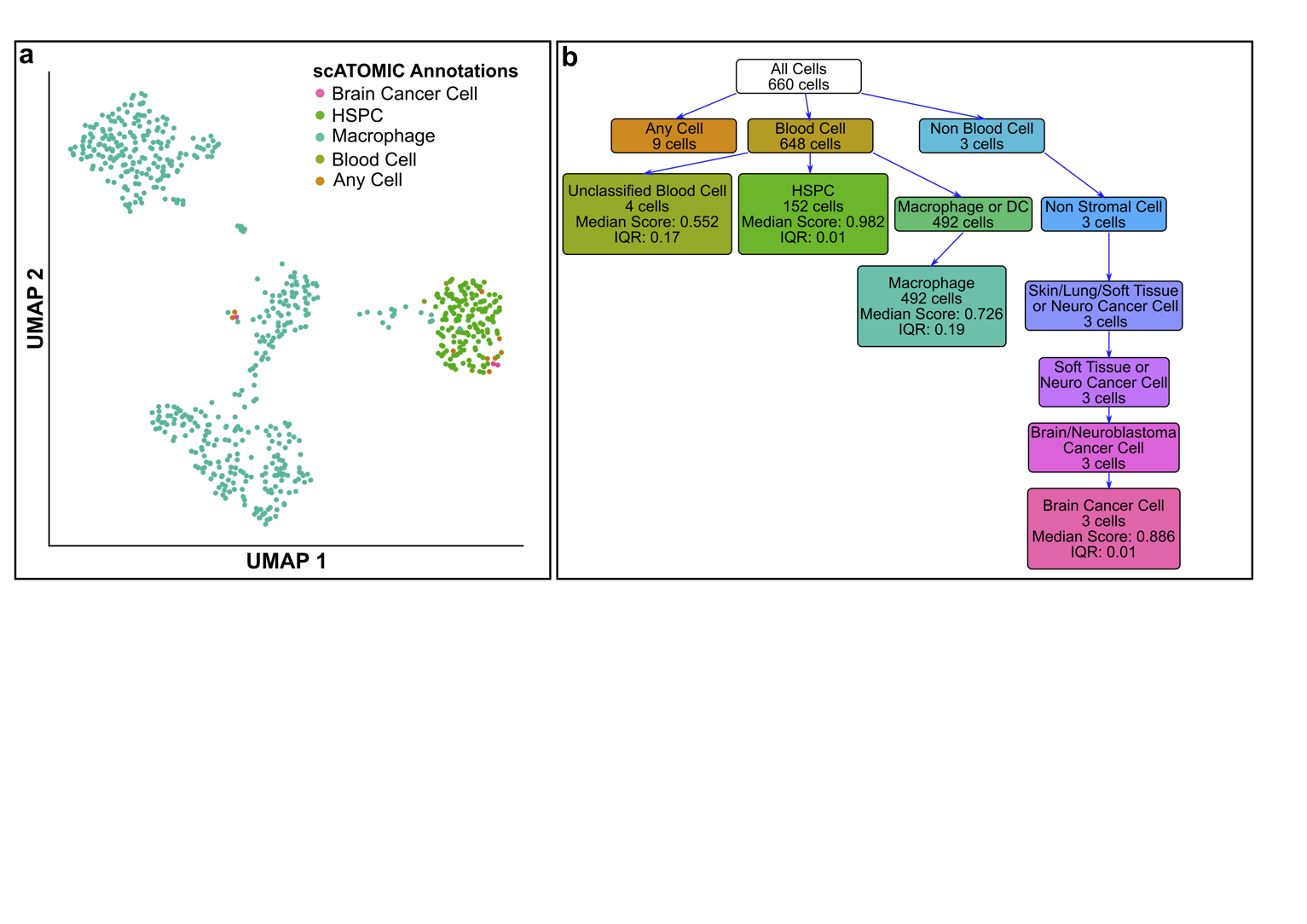


**Supplementary Fig. 10 scATOMIC identifies HSPCs in glioblastoma**. We applied scATOMIC to a dataset of CD45+/CD34+ bead enriched cells from primary glioblastoma tissue. **a,** UMAP illustration of scATOMIC predictions. **b,** scATOMIC correctly identified HSPCs and separated those from other cell types, including macrophages and 3 brain cancer cells, thus recapitulating the original cancer type reported by the authors^7^.

**
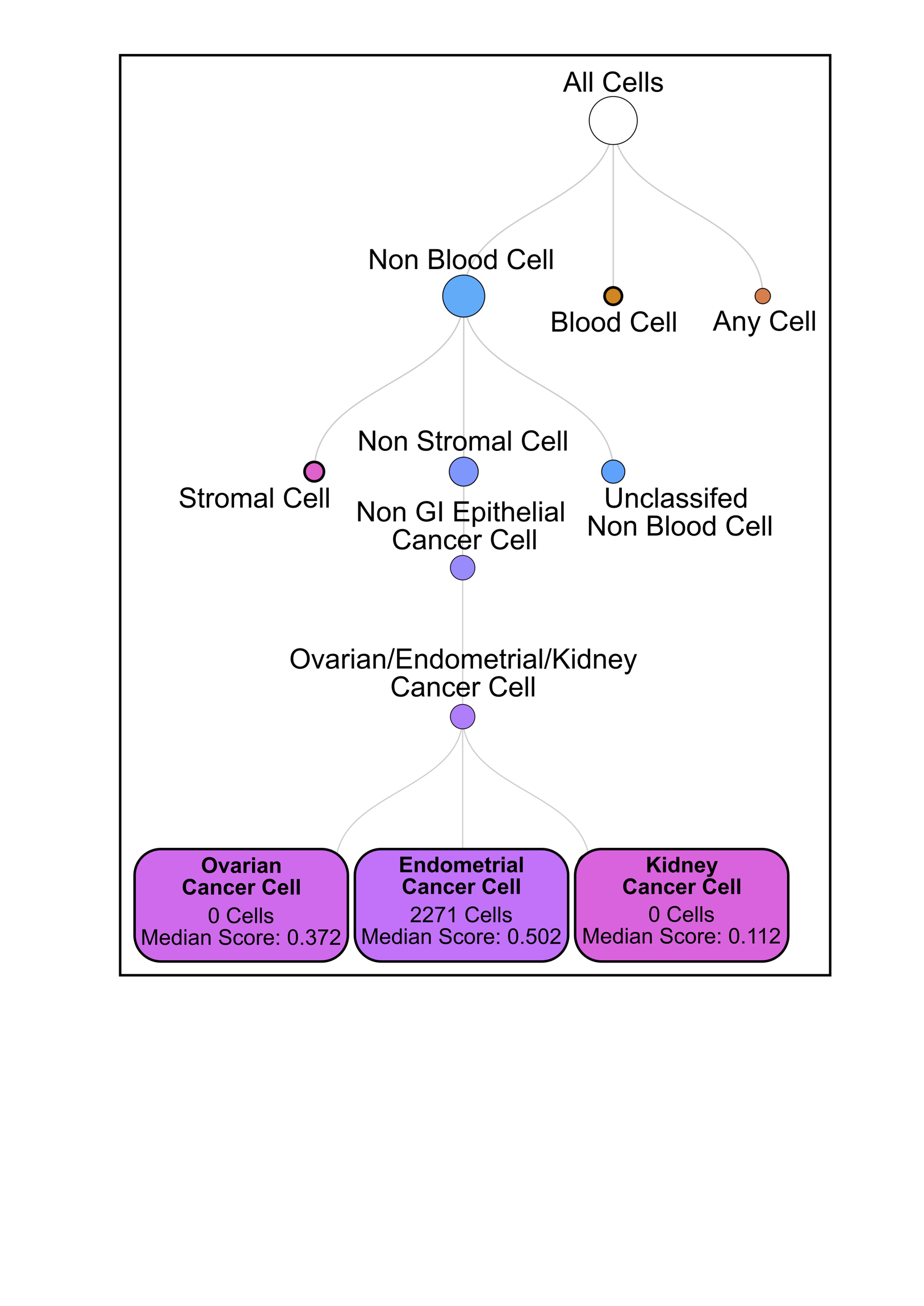
**

**Supplementary Fig. 11. Example for a misclassification of an ovarian cancer specimen.** The classification hierarchy for cancer cells is shown with increasing resolution down the tree. Median classification scores (that is, the proportion of trees voting for each class), and the resulting number of cells are shown for kidney, endometrial, and ovarian cancer cells in the final branch.


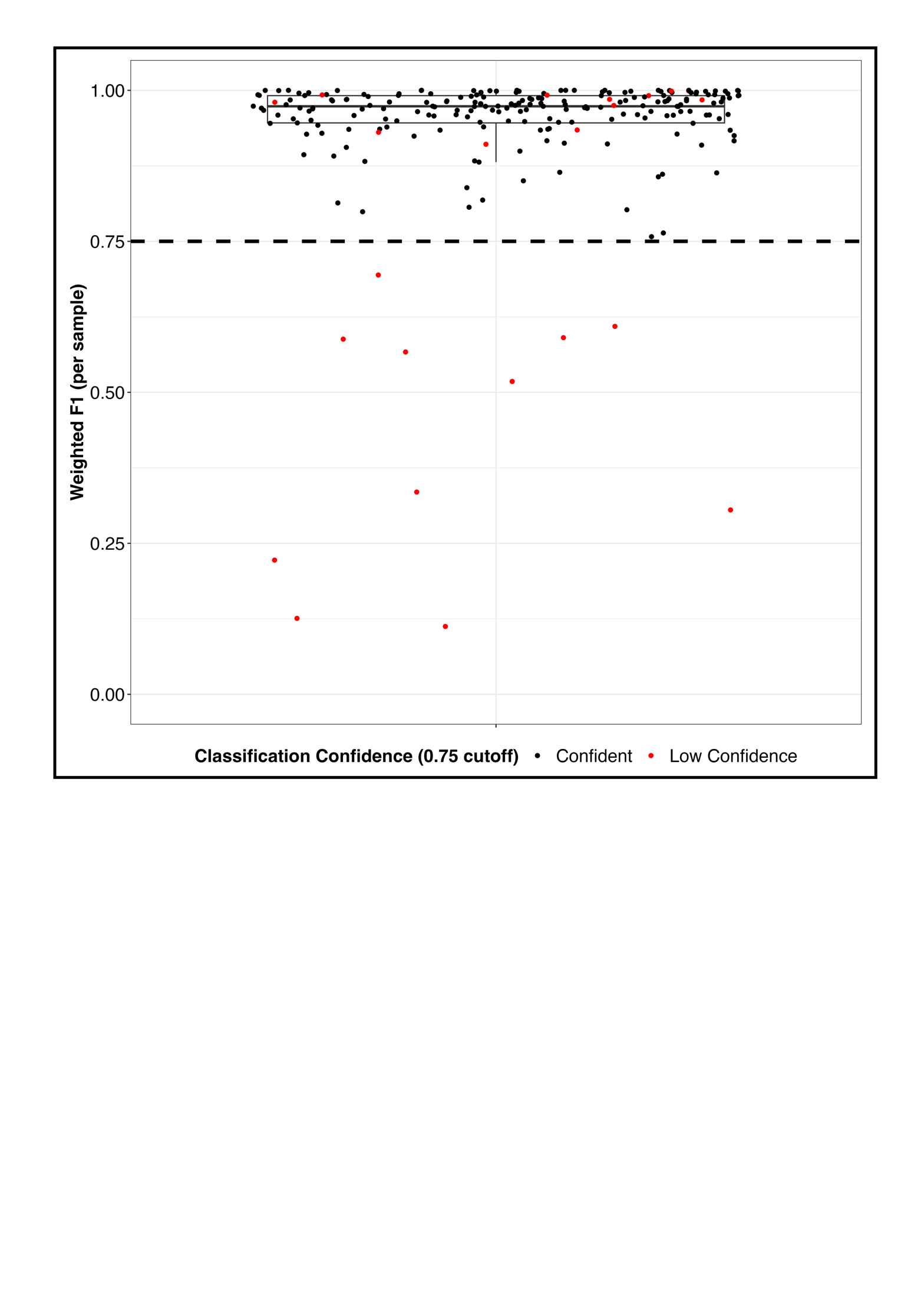


**Supplementary Fig. 12. Inter-sample variability in scATOMIC performance.** Weighted F1 scores are plotted for each sample in the external validation dataset (n=225). Each sample (dot) is coloured by its assigned classification confidence status derived from the proportion of confidently annotated cells. All of the samples with a weighted F1 score < 0.75 also had less than 75% of cells obtaining high confident annotations. Boxes and whiskers represent the lower fence, first quartile (Q1), median (Q2), third quartile (Q3), and upper fence. Source data are provided as a Source Data file.

**Supplementary Note 1 – Query of unrepresented cell types:**


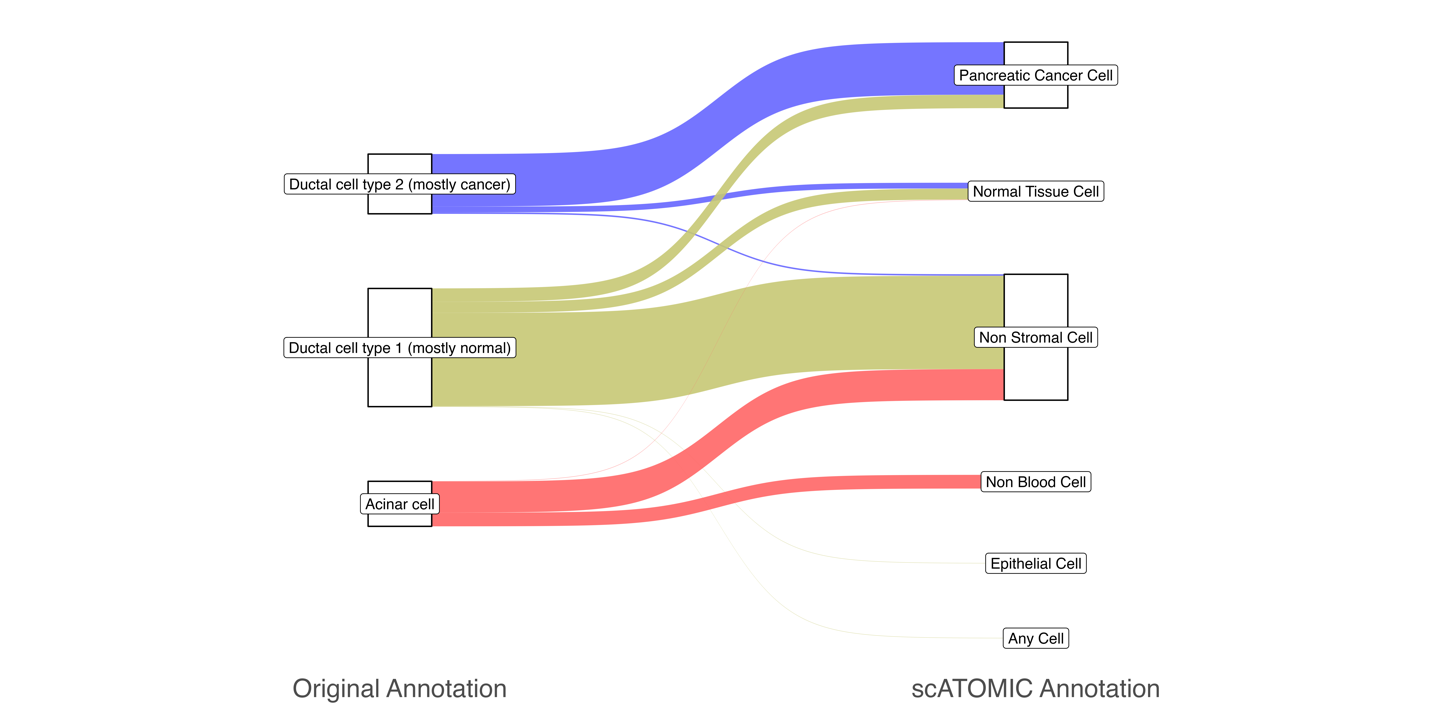
Here we demonstrate in detail an example of how scATOMIC classifies query cell types that are not within its reference (Dec. 2022). In the context of tumour microenvironments these cells most likely correspond to non-malignant cells that are unique to the tissue such as acinar and ductal cells in the pancreas. In this example, as shown below in Supplementary Note 1 Fig. 1, scATOMIC annotated acinar cells and most normal ductal cells with correct intermediate labels of “non-blood cells” and “non stromal cells” differentiating them from cancer cells.

**Supplementary Note 1 Fig. 1. Sankey plot of original annotations compared to scATOMIC annotations in pancreatic ductal cells.** Pancreatic ductal cells from patient T13 in Peng et al^8^ were classified with scATOMIC using default settings. Colours represent original author derived annotations.

This is done as cells pass through the classification layers of the hierarchy (Main Fig. 1a). In the first layer of classification, acinar and ductal cells (as determined by the original cell labels associated with this dataset) received IGS_non-blood_ greater than the automatically determined threshold (Supplementary Note 1 Fig. 2). Therefore, they were assigned the intermediate class “non-blood cell” for classification node 1.


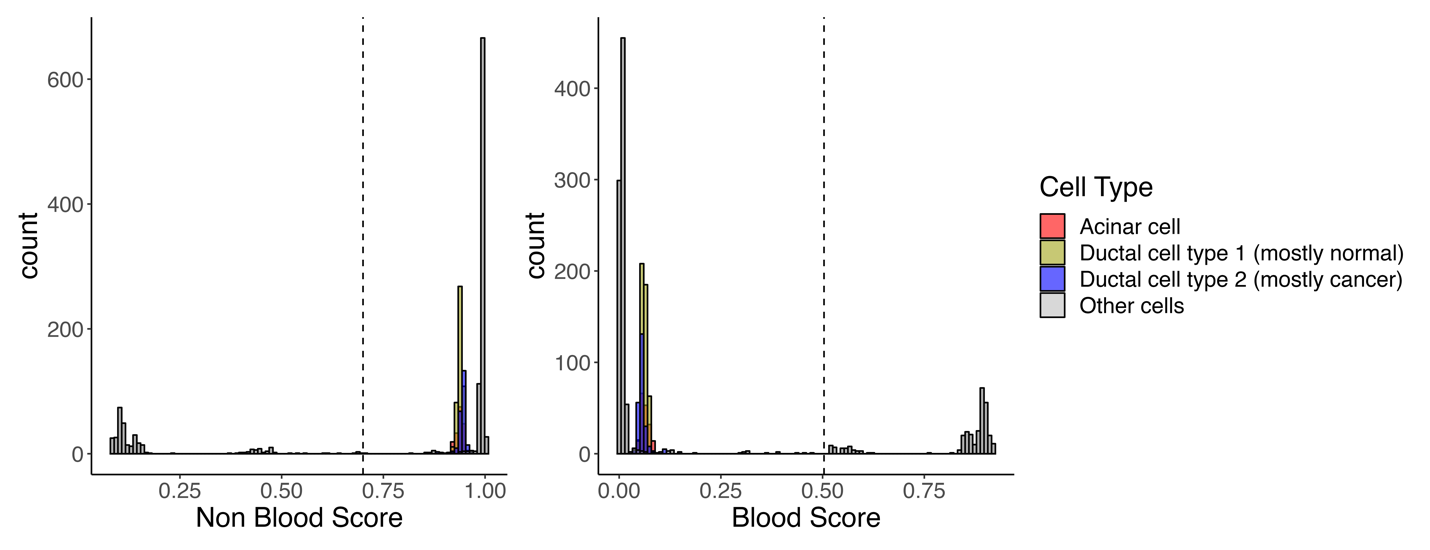
**First classification:**

**Supplementary Note 1 Fig, 2. IGS distributions across pancreatic cells at the first classification node of scATOMIC.** The dashed line represents the cutoff for a confident IGS for classification. All cells received confident scores.

In the second classification node, a fraction of those acinar cells (within red box) did not receive IGS_non-stromal_ greater than the threshold thus remained with a final classification of non-blood cell (Supplementary Note 1 Fig. 3). The remaining cells were intermediately annotated as non-stromal and progressed to the third classification node.

**Second classification:**


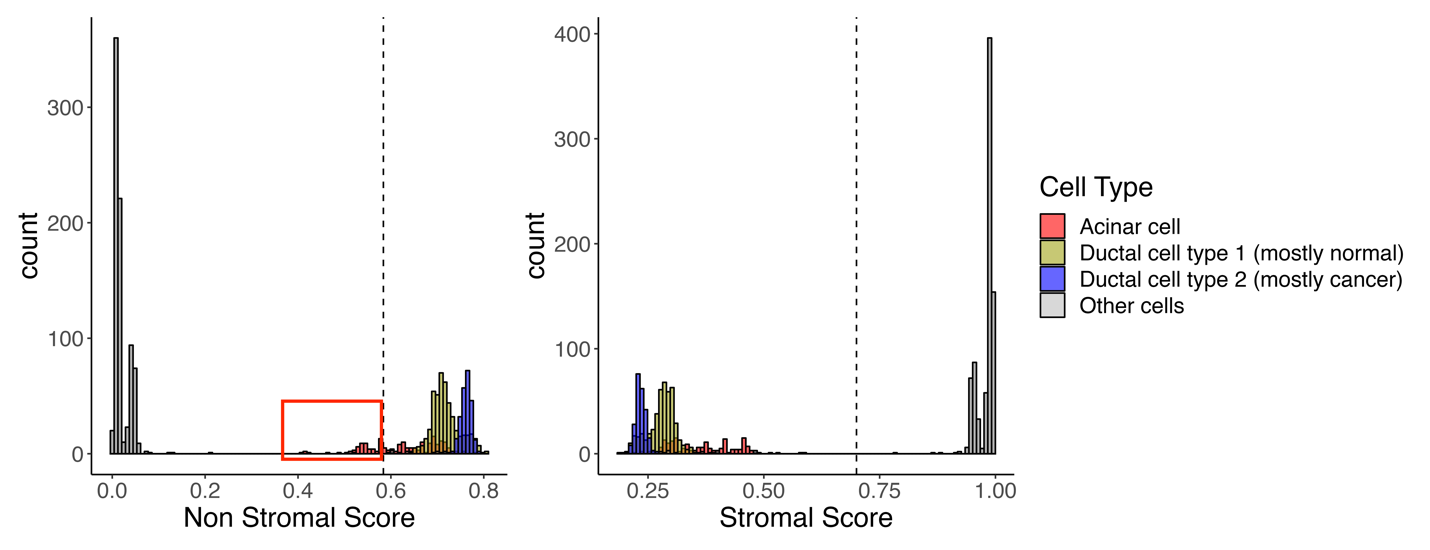


**Supplementary Note 1 Fig. 3. IGS distributions across non-blood pancreatic cells at the second classification node of scATOMIC.** The dashed line represents the cutoff for a confident IGS for classification. Cells in the red box did not receive a confident IGS and are not further classified.

In the third classification task, most of the remaining acinar cells and normal ductal cells (red box, **, Supplementary Note 1 Fig. 4**) did not receive IGS greater than any of the 3 thresholds related to the 3 cancer group classification nodes (Showing only Group 1 cancers below, as an example) thus remained with a final classification of non-stromal cell. Nonetheless, a small subset of ductal cells (green box, **Supplementary Note 1 Fig. 4**) received a confident non-GI epithelial cancer score while the rest of the Ductal cells progressed through the hierarchy and received a confident final cancer annotation (not shown). Next, through the create_summary_matrix() function (see Supplementary Note 2), pancreatic cancer signatures were assessed in this small subset of ductal cells with intermediate cancer annotation (green box).

**Third classification:**


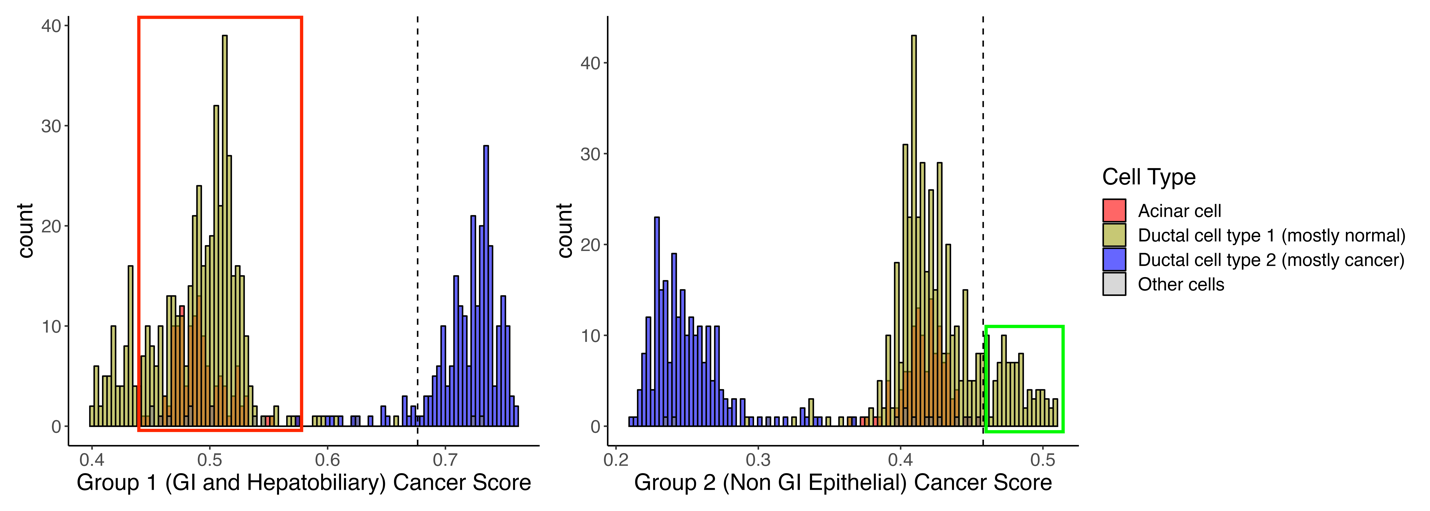


**Supplementary Note 1 Fig. 4. IGS distributions across non-stromal pancreatic cells at the third classification node of scATOMIC.** The dashed line represents the cutoff for a confident IGS for classification. Cells in the red box did not receive a confident IGS and are not further classified. Cells in the green box received a confident IGS for the incorrect cancer type. These cells have their labels converted to the correct cancer type using the create_summary_matrix() function.

Within this subset, Ductal cells that clustered based on their high pancreatic cancer signature with the scATOMIC annotated pancreas cancer cells received a final label of pancreatic cancer, while Ductal cells that had normal pancreatic signature received a final label of normal tissue cell (As Illustrated in main Fig. 1f).

**Supplementary Note 2 – Schematic and description of scATOMIC main functions:**

**
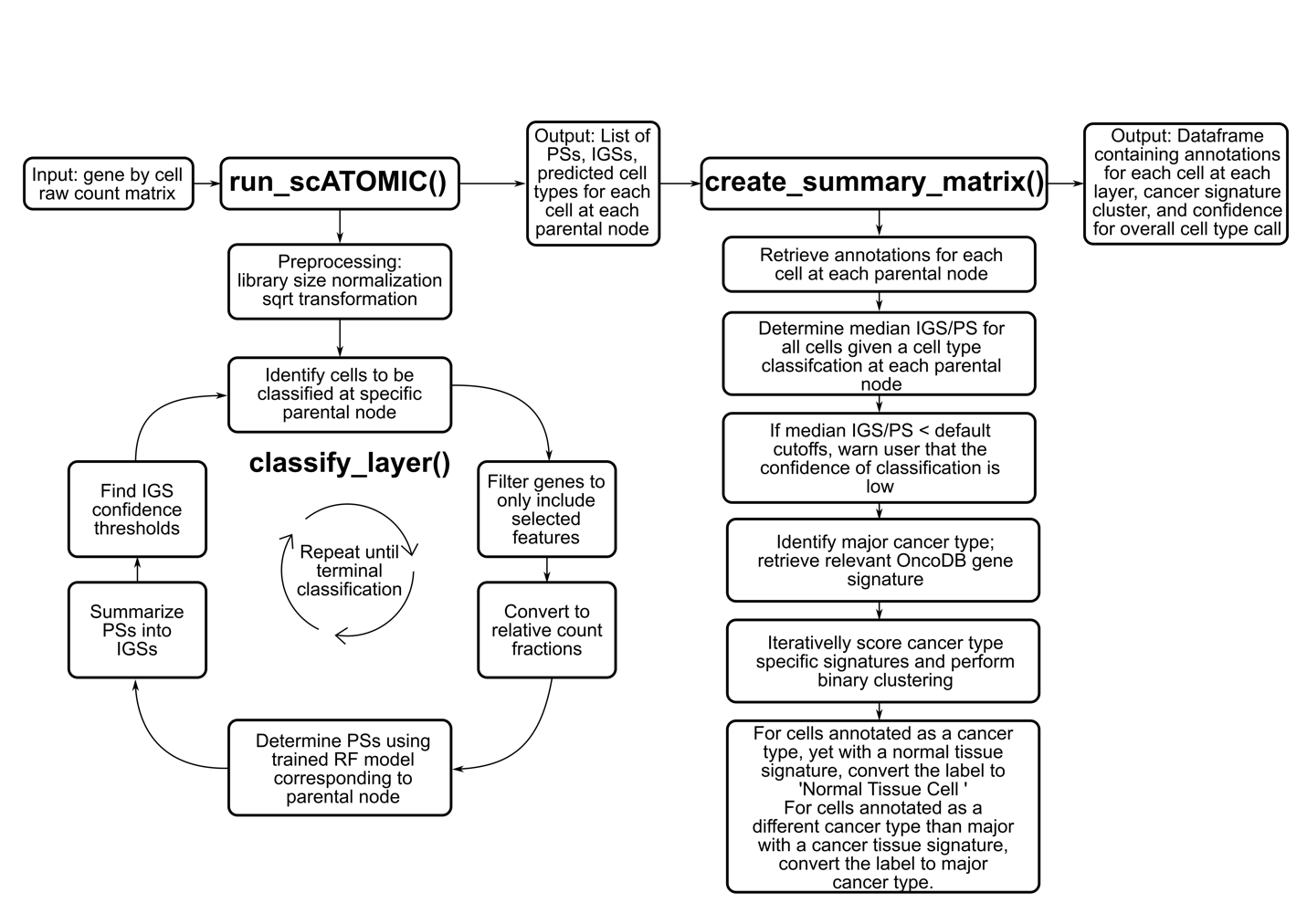
**

**Supplementary Note 2 Fig. 1. Schematic of the main functions of scATOMIC.** The main functions of scATOMIC are described.

**run_scATOMIC():** The user inputs a raw, unnormalized gene by cell count matrix. Library size normalization and square root transformation on the entire gene by cell count matrix is performed. Then, cells are classified through stepwise classification. run_scATOMIC identifies the cells to be classified at each layer and calls the classify_layer() function on them. classify_layer() is automatically provided with the necessary inputs for classification at each node including features selected for classification, the pretrained model, and a list of cell indices to be classified at each node. classify_layer() returns prediction scores (corresponding to the proportion of tree voting for each terminal class) using the random forest models, summarizes PSs into IGSs, and determines IGS confidence thresholds. This process is repeated until all cells are given their terminal classification. The output of run_scATOMIC() is a list of PSs, IGSs, and the relevant predicted cell classes for each cell across the hierarchy.

**create_summary_matrix():**The user inputs the list returned from run_scATOMIC() and the raw count matrix. Annotations at each parental node are retrieved from the list and summarized into a data frame. Median IGS (or PSs in terminal nodes) for each cell type that was classified are also added to the data frame. Using pre-determined cut-offs for the median IGS/PSs, the user is warned if a classification is of low confidence. The cancer type is identified by the label that was assigned to the majority of cells and using the OncoDB gene signatures, cancer signatures are scored in each cell. True cancer cells are identified through iterative clustering and normal cell filtering until only cancer cells remain. Cells which received a cancer annotation in run_scATOMIC but are not within the cancer cluster are converted to Normal Tissue Cell. Cells which receive a different cancer label yet express major cancer type signatures have their labels converted to the major cancer type. The final output is a data frame containing cell barcodes, the annotations of each cell at each layer, the cancer or normal prediction based on the cancer signature scoring, and confidence flag.

**References**

1. Kim, N. *et al.* Single-cell RNA sequencing demonstrates the molecular and cellular reprogramming of metastatic lung adenocarcinoma. *Nat. Commun. 2020 111* **11**, 1–15 (2020).

2. Chen, Z. *et al.* Single-cell RNA sequencing highlights the role of inflammatory cancer-associated fibroblasts in bladder urothelial carcinoma. *Nat. Commun.* **11**, 1–12 (2020).

3. Chen, S. *et al.* Single-cell analysis reveals transcriptomic remodellings in distinct cell types that contribute to human prostate cancer progression. *Nat. Cell Biol.* **23**, 87–98 (2021).

4. Dong, R. *et al.* Single-Cell Characterization of Malignant Phenotypes and Developmental Trajectories of Adrenal Neuroblastoma. *Cancer Cell* **38**, 716-733.e6 (2020).

5. Couturier, C. P. *et al.* Single-cell RNA-seq reveals that glioblastoma recapitulates a normal neurodevelopmental hierarchy. *Nat. Commun. 2020 111* **11**, 1–19 (2020).

6. Slyper, M. *et al.* A single-cell and single-nucleus RNA-Seq toolbox for fresh and frozen human tumors. *Nat. Med.* **26**, 792–802 (2020).

7. Lu, I. N. *et al.* Tumor-associated hematopoietic stem and progenitor cells positively linked to glioblastoma progression. *Nat. Commun. 2021 121* **12**, 1–16 (2021).

8. Peng, J. *et al.* Single-cell RNA-seq highlights intra-tumoral heterogeneity and malignant progression in pancreatic ductal adenocarcinoma. *Cell Res. 2019 299* **29**, 725–738 (2019).
