# Supplementary material for: Pan-cancer classification of single cells in the tumour microenvironment
Source: Nat Commun. 2023 Mar 23;14:1615. doi: 10.1038/s41467-023-37353-8 (PMC10036554; doi:10.1038/s41467-023-37353-8)
Supplement: Supplementary file 3 — Description to Additional Supplementary Information [file 41467_2023_37353_MOESM3_ESM.pdf]

Description of Supplementary Data

Supplementary Data 1 – Reference datasets used to train scATOMIC's core cell class models. For each cell type in the reference the dataset source, number of cells, and mean F1 score in k-fold cross validation is shown.

Supplementary Data 2 – List of the cell lines from *Kinker, G.S. et al (2020)* used in the reference dataset. For each cell line, the number of cells, cancer type, and cancer subtype is shown.

Supplementary Data 3 – Pan-cancer external validation datasets. For each sample the source of data, condition, cell types, and number of cells is shown. F1 scores for scATOMIC, scmap-cell, SingleR, SingleCellNet, CHETAH, Seurat, and scType are shown for each cell type from each sample.

Supplementary Data 4 – Training set samples for breast cancer subclassification. Immunohistochemical subtype and the number of cells is shown for each sample.

Supplementary Data 5 –Validation breast cancer samples from *Pal, et al (2021)*. For each sample, the reported subtype, predicted subtype, and cell composition is shown.

Supplementary Data 6 – List of annotated metastatic samples used for tumour tissue of origin prediction.

Supplementary Data 7 – List of features being used by scATOMIC for cell classification. Features are ordered to indicate genes that differentiate one cell type from all the other cells represented in the same layer.

Supplementary Data 8 – Accession numbers for datasets used across manuscript and platforms that were used.
